# Changing epidemiology and antimicrobial resistance of bacteria causing bacteremia in Taiwan: 2002–2020

**DOI:** 10.1128/spectrum.00608-24

**Published:** 2024-06-25

**Authors:** Ying-Chi Huang, Shu-Chen Kuo, Chi-Tai Fang, Tsai-Ling Lauderdale

**Affiliations:** 1 National Institute of Infectious Diseases and Vaccinology, National Health Research Institutes, Zhunan, Taiwan; 2 Institute of Epidemiology and Preventive Medicine, College of Public Health, National Taiwan University, Taipei, Taiwan; 3 Division of Infectious Diseases, Department of Internal Medicine, National Taiwan University Hospital, Taipei, Taiwan; Taichung Veterans General Hospital, Taichung, Taiwan

**Keywords:** bacteremia, antimicrobial resistance, Taiwan, vancomycin-resistant *Enterococcus*, third-generation cephalosporin resistance

## Abstract

Bacteremia is associated with significant morbidity and mortality. The emergence of bacteria with antimicrobial resistance (AMR) has further exacerbated the poor outcomes associated with bacteremia. The Taiwan Surveillance of Antimicrobial Resistance (TSAR) program was established in 1998 to monitor bacterial epidemiology and antimicrobial resistance trends across all patient types and age groups. Between 2002 and 2020, a total of 14,539 non-duplicate bacteremia isolates were collected biennially from 29 hospitals during the months of July–September as part of the TSAR program. The three most common bacteremia agents were *Escherichia coli* (31%), *Staphylococcus aureus* (13.6%), and *Klebsiella pneumoniae* (12.7%) overall. However, there was a steady increase in the proportions of *E. coli* and *Enterococcus faecium* isolated from bacteremia cases (both *P* < 0.001), while the proportions of *Acinetobacter* spp. decreased. Regarding antimicrobial resistance, there was a notable increase in rates of third-generation cephalosporin and fluoroquinolone non-susceptibility among *E. coli* and *K. pneumoniae*, while the rates of carbapenem non-susceptibility were elevated but remained milder in these two species, especially in *E. coli*. Of concern is the alarming increase in vancomycin resistance among *E. faecium*, rising from 10.0% in 2004 to 47.7% in 2020. In contrast, the prevalence of methicillin-resistant *S. aureus* has remained stable at 51.2% overall. In conclusion, *E. coli*, with increasing third-generation cephalosporin and fluoroquinolone resistance, is the predominant cause of bacteremia in Taiwan during the 18-year surveillance. The escalating proportion of *E. faecium* in bacteremia, coupled with a concurrent upsurge in vancomycin resistance, presents a therapeutic challenge in the recent decade.

**IMPORTANCE:**

AMR surveillance not only enables the identification of regional variations but also supports the development of coordinated efforts to combat AMR on a global scale. The TSAR has been a biennial, government-endorsed, multicenter study focusing on pathogens isolated from inpatients and outpatients in Taiwan hospitals since 1998. Our report presents an 18-year comprehensive analysis on blood isolates in the 2002–2020 TSAR program. The study highlights an alarming increase in the proportion of *E. faecium* causing bacteremia accompanied by elevated vancomycin resistance. It is worth noting that this trend differs from the observations in the United States and China. Understanding the composition of bacteria causing bacteremia, along with their prevalence of antimicrobial resistance, holds significant importance in establishing healthcare and research priorities. Additionally, this knowledge serves as a critical factor in evaluating the effectiveness of preventive interventions.

## INTRODUCTION

Bacteremia is a severe infection that causes significant comorbidity and mortality (1). The pathogens associated with bacteremia vary among countries and over time (2–4). Additionally, the changing rates of antimicrobial resistance among bacteria causing bacteremia can impact the choice of appropriate antibiotics for patient treatment. Therefore, continuous surveillance of antimicrobial resistance in bacteremia is crucial for every country to establish local data (5–8).

In a recent list published by the World Health Organization, critical and high priority antimicrobial-resistant bacteria with urgent research and discovery for new drugs include Gram-positive bacteria (high priority) such as methicillin-resistant *Staphylococcus aureus* (MRSA) and vancomycin-resistant *Enterococcus faecium* (VRE), and Gram-negative bacteria like carbapenem-resistant and third-generation cephalosporin-resistant *Enterobacterales* (9). These antimicrobial-resistant pathogens have been associated with high mortality rates in previous studies (10–12).

The Taiwan Surveillance of Antimicrobial Resistance (TSAR) program has been monitoring the prevalence of antimicrobial resistance since 1998 (13, 14). The TSAR program is a longitudinal, multicenter, hospital-based national surveillance of clinical isolates from all sample types and patient groups (15–17). The surveillance data are provided on the website (https://infection.nhri.edu.tw/), created by the National Health Research Institutes, which aims to provide accessible and comprehensive information on infectious diseases, antibiotic consumption, and antimicrobial resistance to the government, scientific society, medical community, and the general public. This study aims to evaluate the epidemiology and antimicrobial resistance of pathogens causing bacteremia from 2002 to 2020 using the TSAR collection to ensure appropriate treatment.

## MATERIALS AND METHODS

### Isolate collection, species identification, and data analysis

Isolates were collected as part of the TSAR program biennially from July to September between 2002 (TSAR III) and 2020 (TSAR XII). The isolates were obtained from a total of 29 hospitals distributed across all four regions of Taiwan, including 12 medical centers and 17 regional hospitals. For the regular collection, each hospital was asked to first collect 200 isolates sequentially without specifying the species and specimen types, and included 50 outpatient isolates, 30 adult intensive care unit (ICU) and 100 adult non-ICU inpatient isolates, and 20 pediatric isolates. After the aforementioned collection, another 20 (2002–2008) and 50 (2010–2020) isolates recovered from blood or sterile body sites were collected. After the above regular collection, special collection on certain species was carried out. Further details regarding the collection protocol are provided in the supplemental materials. For this study, only isolates from blood were analyzed, with focus on changes in their ranking and prevalence of antimicrobial susceptibility. The study period was categorized as 2002–2010 and 2012–2020.

Demographic data on isolates, including patient age, specimen type, and the unit from which isolates were collected (ICU, non-ICU, and outpatient including emergency room), were provided by the hospitals. Age groups were identified as pediatrics (aged <18 years old), adults (18–64 years old), and elderly (≥65 years old). The isolates were collected from clinical samples as part of standard care. Matrix-assisted laser desorption-ionization time-of-flight mass spectrometry (Bruker Daltonik, Germany) and VITEK system and conventional biochemical reactions (as needed) were used for species identification.

### Antimicrobial susceptibility testing

Results of antimicrobial susceptibility testing of the overall top 10 ranked pathogens from blood samples were analyzed in this study (details listed in Table S1). Minimum inhibitory concentrations (MICs) were determined by broth microdilution using Sensititre standard and custom-designed panels (Trek Diagnostics, East Grinstead, UK). The antimicrobial susceptibility breakpoints were applied in alignment with the newest Clinical and Laboratory Standards Institute M100 recommendation (18). In this study, the criterion that defines non-susceptibility to carbapenem is an imipenem MIC of >1 µg/mL for *Enterobacterales* and >2 µg/mL for *Pseudomonas aeruginosa* and *Acinetobacter* spp. Third-generation cephalosporin-non-susceptible *Enterobacterales* were defined as a cefotaxime MIC of >1 µg/mL. The study also applied the newly revised piperacillin-tazobactam and amikacin clinical breakpoints for *Enterobacterales* (18).

### Statistical analysis

Antimicrobial susceptibility was analyzed using WHONET software (19). A χ^2^ test was conducted to analyze the proportion of bacterial species between 2002–2010 and 2012–2020. Cochran-Armitage trend test was performed to determine whether an overall trend was observed in the proportion over time. A *P* value less than 0.05 was considered statistically significant. All analyses were performed using SPSS Statistics version 22 (IBM Corp., Armonk, NY, USA).

## RESULTS

### Distribution of clinical bacteremia isolates

From 2002 to 2020, a total of 14,539 bacterial isolates from blood were enrolled in the TSAR program. Among these, half or 55.0% (*n* = 7,997) were from elderly patients aged 65 years old or more; 38.1% (*n* = 5,545) were from adults aged between 18 and 64 years old; 4.6% (*n* = 6,663) were from children aged less than 18 years old; and the remaining 334 isolates had no data regarding age. Half or 49.5% of the isolates were from medical centers (*n* = 7,192). A total of 36.5%, 26.2%, and 25.3% of the isolates were from hospitals in central, northern, and southern Taiwan, respectively, and the remaining 12.0% were from hospitals in the least populated eastern Taiwan. Among isolates with available patients’ hospital location records, 40.6% (*n* = 5,803) were from outpatients (including the emergency room); 45.4% (*n* = 6,496) were from non-ICU inpatients; and 14% (*n* = 2,002) were from patients in the ICU.

Ranking via isolates from 2012 to 2020, we found that the overall top 10 bacteria causing bacteremia were *Escherichia coli* (32.3%), *Staphylococcus aureus* (13.3%), *Klebsiella pneumoniae* (12.6%), *Pseudomonas aeruginosa* (4.9%), *Acinetobacter* spp. (3.2%), *Enterobacter* spp. (2.9%), *Salmonella* spp. (2.6%), *Enterococcus faecalis* (2.1%), *E. faecium* (1.8%), and group B *Streptococcus* (GBS) (1.8%) among all patients (Table 1). The ranking differed between 2002-2010 and 2012–2020, mainly attributed to the increased proportion of *E. faecium* isolates during 2012–2020.

**TABLE 1:** Top 10 pathogens causing bacteremia in Taiwan, 2002–2010 vs. 2012–2020

Rank order^*a*^	Species/genus	Total	2002–2010	2012–2020	Rate difference (%)	*P* value	Trend
1	*Escherichia coli*	4,511 (31.0)	1,704 (28.9)	2,807 (32.3)	3.50	<0.001	Increase
2	*Staphylococcus aureus*	1,975 (13.6)	822 (14.0)	1,153 (13.3)	−0.60	0.286	
3	*Klebsiella pneumoniae*	1,844 (12.7)	751 (12.8)	1,093 (12.6)	−0.10	0.851	
4	*Pseudomonas aeruginosa*	672 (4.6)	250 (4.2)	422 (4.9)	0.60	0.072	
5	*Acinetobacter* spp.^*b*^	524 (3.6)	245 (4.2)	279 (3.2)	−0.90	0.003	Decrease
6	*Enterobacter* spp.	443 (3.0)	190 (3.2)	253 (2.9)	−0.30	0.303	
7	*Salmonella* spp.	376 (2.6)	149 (2.5)	227 (2.6)	0.10	0.719	
8	*Enterococcus faecalis*	318 (2.2)	134 (2.3)	184 (2.1)	−0.10	0.554	
9	*Enterococcus faecium*	206 (1.4)	49 (0.8)	157 (1.8)	1.00	<0.001	Increase
10	Group B *Streptococcus*	253 (1.7)	100 (1.7)	153 (1.8)	0.10	0.744	
	Other species^*c*^	3,419 (23.5)	1,499 (25.4)	1,920 (22.2)			
	Total	14,539 (100)	5,892 (100)	8,647 (100)			

^*a*^ The ranking order shown is based on the number of isolates in 2012–2020.

^*b*^ *Acinetobacter* spp., here refers to *A. baumannii, A. nosocomialis*, and *A. pittii*, 3 species of the previously named *Acinetobacter calcoaceticus-A. baumannii* complex.

^*c*^ Other identified species included coagulase-negative *Staphylococcus* spp. (6.6%), viridans *Streptococcus* spp. (2.2%), *Proteus* spp. (1.9%), *Serratia* spp. (1.3%), and *Aeromonas* spp. (1.1%). Species comprising less than 1% are not listed here.

### Changing epidemiology of bacteremia isolates

During the 18-year surveillance period, the proportion of *E. coli* isolates increased from 28.9% to 32.3% between 2002–2010 and 2012–2020 (*P* < 0.001). In contrast, there was a noteworthy decrease of *Acinetobacter* spp. over the surveillance period, from 4.2% to 3.2% (*P* = 0.003). The *Acinetobacter* spp. here refer to isolates belonging to the previously named *Acinetobacter calcoaceticus-Acinetobacter baumannii* complex, including *A. baumannii*, *A. nosocomialis*, and *A*. *pittii*. The composition of *Enterococcus* spp. causing bacteremia changed over time, with a decrease in proportion of *E. faecalis* (from 2.3% to 2.1%, *P* = 0.554) and an increase in proportion of *E. faecium* (from 0.8% to 1.8%, *P* < 0.001) (Table 1; Table S2).

Considering the predominant bacteria among different age groups, we found that *E. coli*, *S. aureus*, and *K. pneumoniae* remained the top three species in both the adult and elderly groups, albeit with different ranking order (Table S3). *K. pneumoniae* was the second predominant bacteria in adult patients, while *S. aureus* was the second predominant bacteria in elder patients. In the pediatric group, the top three bacteria causing bacteremia were *Salmonella* spp., *E. coli*, and *S. aureus*, with *Salmonella* spp. showing a significant increase from 14.2% in 2002–2010 to 24.5% in 2012–2020 (*P* < 0.001). In contrast, the proportion of *Streptococcus pneumoniae* isolates declined from 4.1% in 2002–2010 to 1.9% in 2012–2020 in the pediatric group.

### Antimicrobial non-susceptibility among Gram-negative bacilli

The *in vitro* activities of various antimicrobials against *E. coli*, *K. pneumoniae*, *Enterobacter* spp., *Salmonella* spp., *P. aeruginosa*, and *Acinetobacter* spp., the organisms ranked among the overall top 10 pathogens causing bacteremia, are summarized here with details shown in Table S4.

#### E. coli

**Fig 1:**
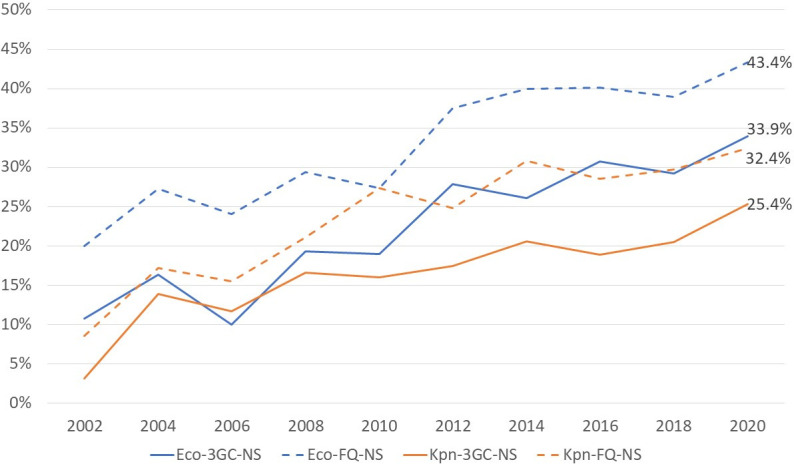
Third-generation cephalosporin and fluoroquinolone non-susceptibility rates among *E. coli* and *K. pneumoniae* isolates causing bacteremia in Taiwan, 2002–2020. Eco, *E. coli*; Kpn, *K. pneumoniae*; 3GC-NS, third-generation cephalosporin-non-susceptible; FQ-NS, fluoroquinolone non-susceptible.

We analyzed 4,431 isolates of *E. coli* collected as part of this study. The test of trend demonstrated increasing non-susceptibility to most β-lactams during the 18-year surveillance period, including cefazolin, cefuroxime, cefotaxime, and cefepime (all *P* < 0.001). The third-generation cephalosporin (3GC) non-susceptibility rate increased from 12% in 2002 to 34.1% in 2020 (Fig. 1). The rate of piperacillin/tazobactam non-susceptibility remained low (3.3%–6.3%), as well as that of imipenem non-susceptibility (0%–1.8%), during the surveillance period. Regarding non-β-lactams, ciprofloxacin non-susceptibility increased from 20% in 2002 to 43.4% in 2020 (*P* < 0.001, Fig. 1). However, non-susceptibility to trimethoprim/sulfamethoxazole decreased from 59.6% in 2002 to 40.8% in 2020 (*P* < 0.001).

#### K. pneumoniae

A total of 1,813 *K*. *pneumoniae* isolates were tested for antimicrobial susceptibility. An increasing proportion of non-susceptibility to most β-lactams, including cefazolin, cefuroxime, cefotaxime, and cefepime (all *P* < 0.001), was observed. The rate of 3GC non-susceptibility among *K. pneumoniae* isolates increased from 3.1% in 2002 to 25.8% in 2020, and non-susceptibility to ciprofloxacin also increased from 8.6% in 2002 to 32.4% in 2020 (*P* < 0.001). The rate of piperacillin/tazobactam non-susceptibility over time remained stable in *K. pneumoniae* (9.7%–15.0%). In contrast to *E. coli*, the rate of non-susceptibility to trimethoprim/sulfamethoxazole increased in *K. pneumoniae* (*P* = 0.004). Of note, the imipenem non-susceptibility of *K. pneumoniae* isolates elevated from 1.6% in 2002 to 8.2% in 2010, and declined in 2018 (1.0%) and 2020 (4.7%).

#### *Enterobacter* spp.

*Enterobacter* spp. demonstrated a stable rate of non-susceptibility to third- and fourth-generation cephalosporins (cefotaxime and cefepime). Interestingly, increasing non-susceptibility to piperacillin/tazobactam, from 8.8% in 2006 to 21.4% in 2020, was noted (*P* < 0.001, Table S4). The imipenem non-susceptibility was high during 2002–2006 (12.5%–15.4%) but decreased in 2010–2014 (0%–3.3%) and then was elevated again in 2020 (14.3%).

#### *Salmonella* spp.

During the 18-year surveillance, nearly all of the *Salmonella* strains were non-typhoidal *Salmonella* (NTS); only four strains were *Salmonella* Typhi (1%, 4 of 376 strains). Due to limited resources, antimicrobial susceptibility tests were not conducted in 2004. There was no imipenem resistance among NTSs in the isolates tested. Although the 3GC non-susceptibility rate among NTS was low during 2004–2016 (0%–3.6%), it soared to 11.9% in 2018 and 9.8% in 2020 (*P* = 0.001). In contrast, the rate of ciprofloxacin non-susceptibility decreased from 36.2% in 2002 to 12.2% in 2020 (*P* = 0.004).

#### P. aeruginosa

The overall non-susceptibility of *P. aeruginosa* to ceftazidime, cefepime, piperacillin/tazobactam, ciprofloxacin, and amikacin remained at less than 20%. The overall non-susceptibility to imipenem was 15.3% but fluctuated over time, reaching 22.8% in 2014, then decreased to 17.5% in 2020.

#### *Acinetobacter* spp.

Imipenem non-susceptibility increased sharply during the surveillance period, rising from 3.0% in 2002 to 45.7% in 2020 (*P* < 0.001). For most of the antibiotics tested, including ceftazidime, cefepime, piperacillin-tazobactam, amikacin, and ciprofloxacin, a significant decrease in non-susceptibility rates was observed over time (all *P* < 0.05). However, the rates of overall non-susceptibility remained high for these antibiotics, ranging from 31.4% to 38.8%. Although the trend in non-susceptibility generally declined from 2002 to 2018, it experienced a resurgence in 2020.

### Antimicrobial non-susceptibility among Gram-positive bacteria

Table S5 lists the antimicrobial non-susceptibility rates of the Gram-positive bacteria that ranked in the overall top 10 pathogens causing bacteremia in this study, which included *S. aureus*, *E. faecalis*, *E. faecium*, and group B *Streptococcus*.

#### S. aureus

**Fig 2:**
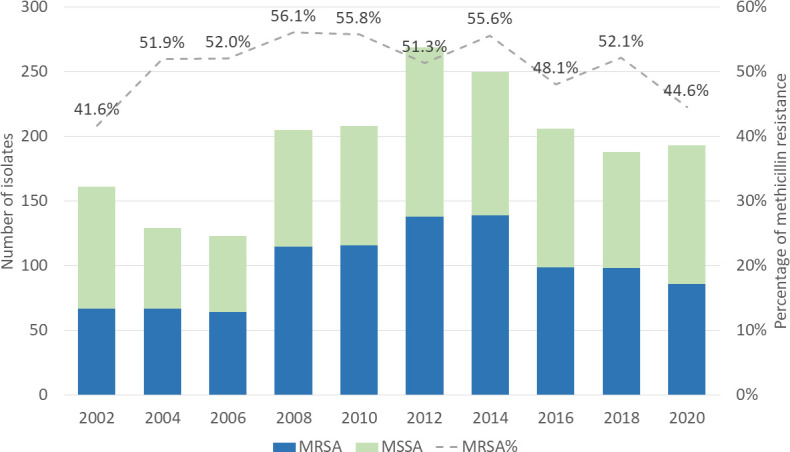
Methicillin resistance rate among *Staphylococcus aureus* isolates causing bacteremia in Taiwan, 2002–2020. MRSA, methicillin-resistant *S. aureus;* MSSA, methicillin-susceptible *S. aureus*.

The overall prevalence of MRSA was 51.2% during the past 18 years. The prevalence of MRSA increased until 2006–2008 and then declined gradually (Fig. 2). The trend analysis revealed no significant change in the proportion of methicillin resistance among *S. aureus*. However, non-susceptibility to clindamycin, rifampin, trimethoprim/sulfamethoxazole, and tetracycline decreased year by year (all *P* < 0.001). The vancomycin and daptomycin non-susceptibility rates were low during the surveillance period (0%–1.9%).

#### *E. faecalis* and *E. faecium*

The overall rates of ampicillin and vancomycin non-susceptibility remained low among *E. faecalis* (0.4%, Table S5), but there has been a significant increase in linezolid non-susceptibility since 2016 (ranging from 11.1% to 12.0%, *P* < 0.001). Before 2016, daptomycin non-susceptibility was around 0%–3.3%, but elevated daptomycin non-susceptibility was noted in 2016 and 2018 (8.9% and 12.0%, respectively) in *E. faecalis*.

**Fig 3:**
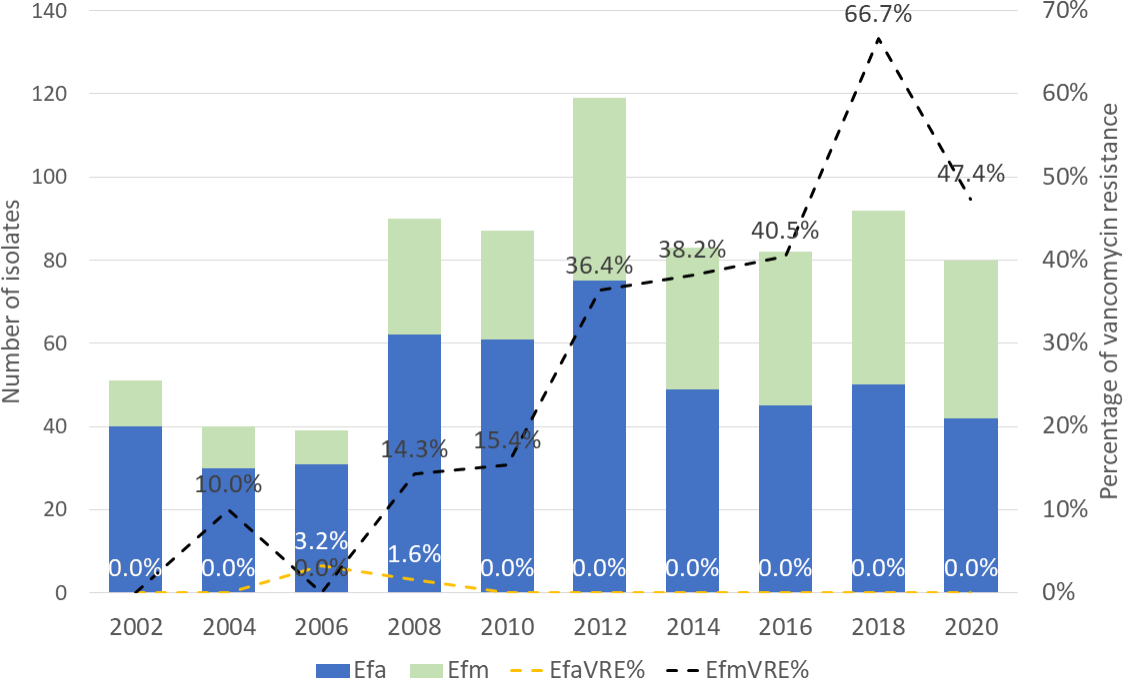
Vancomycin resistance rate among *Enterococcus* isolates causing bacteremia in Taiwan, 2002–2020. Efa, *E. faecalis*; Efm, *E. faecium*; VRE, vancomycin-resistant *Enterococcus*.

In contrast, the rate of vancomycin non-susceptibility among *E. faecium* increased from 14.3% in 2008 to 47.4% in 2020 (Fig. 3). Furthermore, both daptomycin and linezolid non-susceptibility among *E. faecium* also increased in recent years. Daptomycin non-susceptibility was not detected in the *E. faecium* causing bacteremia before 2012 but was found in 2.3%–5.4% of the isolates between 2012 and 2020. Linezolid non-susceptibility was not noted until 2018 (4.8%) and 2020 (2.6%).

#### Group B *Streptococcus* (GBS)

GBS remained susceptible to all β-lactams tested and vancomycin. The rate of levofloxacin non-susceptibility among GBS was 4% overall but fluctuated between 1.0% and 11.1% during 2006–2020. However, the rates of clindamycin, erythromycin, and tetracycline non-susceptibility overall were considerably higher at 36.8%, 41.4%, and 77.5%, respectively.

## DISCUSSION

The TSAR project is a longitudinal surveillance program since 1998 that allows us to monitor pathogens from consecutive bacteremic episodes and testing of all isolates at a central reference laboratory using a standardized protocol. This is the longest and largest-scale surveillance record of changing epidemiology of bacteria causing bacteremia and their antimicrobial resistance in Taiwan.

The major findings from the 18 years of TSAR bacteremia surveillance include

1. Increasing proportion of *E. coli* and decreasing proportion of *Acinetobacter* spp. causing bacteremia from 2002 to 2020.
2. Ongoing increase in third-generation cephalosporin and fluoroquinolone non-susceptibility rates and stable prevalence of carbapenem non-susceptibility among *E. coli* and *K. pneumoniae*.
3. Ongoing increase in the proportion of *E. faecium* along with elevated vancomycin resistance.

This study highlights a significant increase in the proportion of *E. coli* bacteremia cases over the past decade, accounting for one-third (31%) of all cases. This finding is in alignment with similar observations from other regional and national surveillance programs, where the proportion of *E. coli* bacteremia in the Asia-Pacific region was approximately 33.7% (3). Subgroup analysis in our study further revealed that *E. coli* was the leading cause of bacteremia in outpatient departments (41%), inpatient departments (26.4%), and ICUs (15.9%), all showing an upward trend (data not shown). Notably, *E. coli* (31%) accounted for nearly three times the number of bacteremia cases compared to the second most prevalent bacteria, *S. aureus* (13.6%), indicating a significant burden that warrants increased attention.

In addition to the rising proportion of *E. coli* in bacteremia patients, our study also observed an increasing 3GC non-susceptibility in *E. coli* over the years (from 12% in 2002 to 34.1% in 2020, Fig. 2). Similar trends have been reported in the United Kingdom, where 3GC-resistant *E. coli* bacteremia isolates increased significantly from 11.9% to 14.0% (*P* < 0.001) between 2016 and 2020 (6). In the United States, nearly 200,000 extended-spectrum β-lactamase-producing *Enterobacterales* infections occurred per year, which represented an alarming 50% increase over the last half decade (5). Furthermore, the prevalence of ciprofloxacin non-susceptibility in *E. coli* has surged alongside 3GC non-susceptibility in our study (from 20.0% in 2002 to 43.4% in 2020, Fig. 2). Notably, *K. pneumoniae* also exhibited increased rates of 3GC and ciprofloxacin non-susceptibility. Previous studies have identified CTX-M types as the primary resistance genes responsible for 3GC resistance in *E. coli* and *K. pneumoniae* in Taiwan (20, 21). A study by Quan et al. demonstrated potential exchange of plasmids or genetic elements between *E. coli* and *K. pneumoniae*, which may help explain the observed phenomenon in our study (22).

During our 18-year surveillance period, we observed a low rate of imipenem non-susceptibility in *E. coli* (0%–1.8%), but the proportion of carbapenem-non-susceptible *K. pneumoniae* showed a steady increase that peaked in 2016 at 7.4%. This trend aligns with the significant rise of carbapenem-resistant *K. pneumoniae* (CRKP) cases from 2005 to 2017 in China (4). However, starting from 2018, we observed a decline in the proportion of CRKP isolates from blood to below 5%. This decline coincided with an increased emphasis on hospital infection prevention practices and contact isolation, following the publication of infection control guidelines about carbapenem-resistant *Enterobacterales* by the Taiwan Centers for Disease Control in 2017 (version in Chinese, https://www.cdc.gov.tw/File/Get/AsKPxsTxMMfom3CZqcJdJA). While the reasons for this decline are likely multifactorial, the implementation of these infection control measures likely played a significant role.

In contrast to the decline in carbapenem non-susceptibility among *K. pneumoniae*, we observed a significant increase in carbapenem non-susceptibility among *Acinetobacter* isolates, specifically, those belonging to the previously named *A. calcoaceticus-A. baumannii* complex (from 3% in 2002 to 50% in 2020). In the subgroup analysis, the rate of imipenem non-susceptibility was attributed to increased imipenem non-susceptibility in *A. baumannii* (data not shown). A previous study in Taiwan reported increased carbapenem resistance in *A. baumannii* was associated with carriage of bla_OXA-51-like_ and _OXA-23-like_ genes (23).

The overall proportion of *Salmonella* spp. causing bacteremia was similar between 2002–2010 (2.5%) and 2012–2020 (2.6%). Nearly all of the *Salmonella* isolates were NTS (372 out of 376 cases). However, in pediatric patients, there was a significant increase in the proportion of NTS bacteremia, from 14.2% in 2002–2010 to 24.5% in 2012–2020 (*P* < 0.001). Remarkably, among all pediatric patients (< 18 years old), over 98% (125 out of 127) of NTS bacteremia cases occurred in patients less than 5 years old (data not shown). Considering that NTS is a foodborne disease, it is crucial to reinforce food safety education and messaging to parents with children under 5 years old (24).

In contrast, the proportion of *S. pneumoniae* causing bacteremia decreased from 4.1% in 2002–2010 to 1.9% in 2012–2020 in the pediatric group. This change could be attributed to the introduction of national pneumococcal conjugate vaccines (PCV13) catch-up program for all 2- to 5-year-old children that was launched by the National Health Insurance, Ministry of Health and Welfare, in Taiwan since March 2013 and is compatible with findings from our previous study (17).

The prevalence of MRSA in our study reached a plateau in 2008 at 56.1% and then progressively decreased each year to 44.6% in 2020. This trend is consistent with findings from a large global surveillance program, where MRSA occurrences increased from 33.1% in 1997–2000 to a peak of 44.2% in 2005–2008, then subsequently declined to 39.0% in 2013–2016 (25). Vancomycin demonstrated consistently high activity against *S. aureus* in Taiwan, and several newer agents, such as linezolid and daptomycin, exhibited excellent *in vitro* potencies. However, MRSA remains a significant problem and continues to be one of the most common antimicrobial-resistant pathogens encountered in hospitals.

During the period from 2002 to 2020, we observed an increase in the proportion of *E. faecium*. In addition to its increased proportion, nearly half (47.4%) of the *E. faecium* isolated from blood samples were vancomycin resistant in 2020. This finding is particularly concerning when only 7% of *E. faecium* were reported as vancomycin resistant in Taiwan in the 2000s (14). The increase in the proportion of VRE that we report stands in contrast to trends observed in other regional and national surveillance programs, where VRE infections have either declined or remained steady (4, 5). The rise of VRE bacteremia is closely associated with healthcare-associated infections (26). It is likely that the increase in vancomycin resistance is linked to the growing hospitalization of patients undergoing cancer treatment or those with compromised immune systems. These individuals are frequently exposed to antimicrobials for the prevention and treatment of sepsis (26). To address the burden of VRE infections effectively, the importance of antimicrobial stewardship needs to be reinforced (27, 28). Implementing strategies to promote the appropriate use of antimicrobials to prevent the emergence of resistance is vital in combatting the spread of VRE.

This study has some limitations. First, although the data were collected from hospitals in four regions of Taiwan, the TSAR program does not provide population-based information on the incidence of infections. However, it is worth noting that the majority of TSAR hospitals participated in each period of the 18-year surveillance, which enhances the representativeness of the data and provides a comprehensive view of the changing epidemiology of bacteria and antimicrobial resistance in Taiwan. Second, this surveillance did not include rates of mortality and morbidity based on electronic health data from hospitals. Therefore, the impact of these infections on patient outcomes could not be assessed. Future studies incorporating such data would provide valuable insights into the clinical implications of these infections. Additionally, the molecular data pertaining to resistance/virulence genes and strain typing are not presented in this report.

In conclusion, this study contributes to our understanding of the trends in the epidemiology and antimicrobial resistance of bacterial pathogens causing bacteremia in Taiwan. It underscores the importance of ongoing surveillance efforts and the development of effective preventive strategies to combat antimicrobial resistance. Future research should aim to incorporate molecular data with further investigations on the clinical impact of these infections to ultimately guide evidence-based interventions and public health policies.

## SUPPLEMENTAL MATERIAL

Supplemental material spectrum.00608-24-s0001.docx Isolate collection protocol and data analysis; Tables S1 to S5.

ASM does not own the copyrights to Supplemental Material that may be linked to, or accessed through, an article. The authors have granted ASM a non-exclusive, world-wide license to publish the Supplemental Material files. Please contact the corresponding author directly for reuse.
