# Supplementary material for: Changing epidemiology and antimicrobial resistance of bacteria causing bacteremia in Taiwan: 2002–2020
Source: Microbiol Spectr. 2024 Jun 25;12(8):e00608-24. doi: 10.1128/spectrum.00608-24 (PMC11301998; doi:10.1128/spectrum.00608-24)
Supplement: Supplemental material — Isolate collection protocol and data analysis; Tables S1 to S5. [file spectrum.00608-24-s0001.docx]

**Supplementary Materials**

**Isolate collection protocol and data analysis**

For each round of TSAR between 2002 and 2020, isolates were collected biennially by participating hospitals using the following protocol (regular collection): We asked each hospital to first collect 200 isolates sequentially, without specifying the bacterial species and specimen types, to include 50 outpatient isolates, 30 adult ICU and 100 adult non-ICU inpatient isolates, and 20 pediatric isolates. After completion of the above collection, an additional 20 (2002-2006) to 50 (2008-2020) isolates from blood and sterile body sites were collected, again without specifying the species.

After the above regular collection, the hospitals were asked to collect the following pathogens recovered during the remaining collection period (special collection): all *Haemophilus influenzae* and all *Streptococcus pneumoniae* (2002-2020), all group A *Streptococcus* isolates (2004-2020), all group B *Streptococcus* isolates (2010-2014), all but prenatal screening group B *Streptococcus* isolates (2016-2020), and 10 each of *Salmonella* spp., and *Enterococcus* spp., without specifying specimen source.

For the present study, only blood isolates from the regular collection were used for total and subgroup rank order analyses. However, both regular and special collection isolates from blood samples were included in antimicrobial susceptibility testing (AST) analyses of the overall top 10 ranked pathogens. Further details are provided in Supplementary Table S1.

Supplementary Table S1. Summary of isolate collection protocol and data analysis.

| Collection criteria | Year and number of isolates collected/hospital | | | | | | | | | | Included in | |
| --- | --- | --- | --- | --- | --- | --- | --- | --- | --- | --- | --- | --- |
|  | 2002 | 2004 | 2006 | 2008 | 2010 | 2012 | 2014 | 2016 | 2018 | 2020 | Ranking analysis^a^ | AST analysis^b^ |
| Regular collection | | | | | | | | | | | | |
| Any biological samples without specifying the species | 200 | 200 | 200 | 200 | 200 | 200 | 200 | 200 | 200 | 200 | Yes | Yes |
| Blood/sterile samples without specifying the species | 20 | 20 | 20 | 20 | 50 | 50 | 50 | 50 | 50 | 50 | Yes | Yes |
| Special collection (after completion of above regular collection) | | | | | | | | | | | | |
| *H. influenzae* | All | All | All | All | All | All | All | All | All | All | No | No^c^ |
| *S. pneumoniae* | All | All | All | All | All | All | All | All | All | All | No | No^c^ |
| Group A *Streptococcus* |  | All | All | All | All | All | All | All | All | All | No | No^c^ |
| Group B *Streptococcus* |  |  |  |  | All | All | All | All but prenatal screening isolates | | | No | Yes |
| *Salmonella* spp. | 10 | 10 | 10 | 10 | 10 | 10 | 10 | 10 | 10 | 10 | No | Yes |
| *Enterococcus* spp. | 10 | 10 | 10 | 10 | 10 | 10 | 10 | 10 | 10 | 10 | No | Yes |

^a^ Data of ranking analysis on isolates from blood shown in Table 1, Supplementary Table S2, and Supplementary Table S3.

^b^ Data of AST analysis on isolates from blood shown in Supplementary Table S4 and Supplementary Table S5.

^c^ The AST results of *H. influenzae, S. pneumoniae,* and Group A *Streptococcus* are not listed in supplementary Table S4 and S5, respectively, because they were not among the overall top 10 ranked pathogens in this study.

Supplementary Table S2. Pathogens causing bacteremia in Taiwan by year, 2002-2020

| Pathogen | No. (%) | | | | | | | | | | |
| --- | --- | --- | --- | --- | --- | --- | --- | --- | --- | --- | --- |
|  | 2002 | 2004 | 2006 | 2008 | 2010 | 2012 | 2014 | 2016 | 2018 | 2020 | 2002-2020 |
| *E. coli* | 231  (25.0) | 225  (25.6) | 280  (32.1) | 482  (30.2) | 486  (30.0) | 555  (28.2) | 574  (32.8) | 577  (35.6) | 545  (33.1) | 556  (33.5) | 4511  (31.0) |
| *S. aureus* | 151  (16.3) | 131  (14.9) | 123  (14.1) | 208  (13) | 209  (12.9) | 279  (14.2) | 254  (14.5) | 212  (13.1) | 202  (12.3) | 206  (12.4) | 1975  (13.6) |
| *K. pneumoniae* | 117  (12.7) | 122  (13.9) | 105  (12.0) | 202  (12.6) | 205  (12.7) | 226  (11.5) | 218  (12.5) | 222  (13.7) | 214  (13.0) | 213  (12.8) | 1844  (12.7) |
| *P. aeruginosa* | 46  (5.0) | 38  (4.3) | 28  (3.2) | 77  (4.8) | 61  (3.8) | 100  (5.1) | 95  (5.4) | 78  (4.8) | 69  (4.2) | 80  (4.8) | 672  (4.6) |
| *Acinetobacter* spp.^a^ | 32  (3.5) | 28  (3.2) | 36  (4.1) | 86  (5.4) | 63  (3.9) | 78  (4.0) | 62  (3.5) | 49  (3.0) | 56  (3.4) | 34  (2.0) | 524  (3.6) |
| *Enterobacter* spp. | 31  (3.4) | 28  (3.2) | 35  (4.0) | 55  (3.4) | 41  (2.5) | 63  (3.2) | 51  (2.9) | 38  (2.3) | 45  (2.7) | 56  (3.4) | 443  (3.0) |
| *Salmonella* spp. | 19  (2.1) | 24  (2.7) | 30  (3.4) | 35  (2.2) | 41  (2.5) | 54  (2.7) | 55  (3.1) | 35  (2.2) | 41  (2.5) | 42  (2.5) | 376  (2.6) |
| *E. faecalis* | 25  (2.7) | 12  (1.4) | 13  (1.5) | 40  (2.5) | 44  (2.7) | 52  (2.6) | 32  (1.8) | 22  (1.4) | 41  (2.5) | 36  (2.2) | 317  (2.2) |
| *E. faecium* | 7  (0.8) | 3  (0.3) | 4  (0.5) | 19  (1.2) | 16  (1.0) | 30  (1.5) | 25  (1.4) | 31  (1.9) | 37  (2.2) | 34  (2.0) | 206  (1.4) |
| Group B *Streptococcus* | 14  (1.5) | 21  (2.4) | 9  (1.0) | 20  (1.3) | 35  (2.2) | 32  (1.6) | 25  (1.4) | 28  (1.7) | 27  (1.6) | 41  (2.5) | 252  (1.7) |
| Others | 251  (27.2) | 246  (28.0) | 209  (24.0) | 374  (23.4) | 419  (25.9) | 497  (25.3) | 360  (20.6) | 329  (20.3) | 370  (22.5) | 364  (21.9) | 3419  (23.5) |
| Total | 924  (100) | 878  (100) | 872  (100) | 1598  (100) | 1620 (100) | 1966  (100) | 1751  (100) | 1621 (100) | 1647 (100) | 1662  (100) | 14539  (100) |

^a^ *Acinetobacter* spp., here refers to *A. baumannii,* *A. nosocomialis*, and *A. pitti*, 3 species of the previously named *A. calcoaceticus-A. baumannii* complex.

Supplementary Table S3. Pathogens causing bacteremia in different age groups in Taiwan, 2002-2020

| Ranking ^a^ | Species/Genus | 2002-2010 | 2012-2020 | Rate difference | P value | Trend |
| --- | --- | --- | --- | --- | --- | --- |
| Pediatrics |  |  |  |  |  |  |
| 1 | *Salmonella* spp. | 49 (14.2) | 78 (24.5) | 10.3% | <0.001 | Increase |
| 2 | *E. coli* | 28 (8.1) | 33 (10.4) | 2.3% | 0.314 |  |
| 3 | *S. aureus* | 31 (9) | 32 (10.1) | 1.1% | 0.636 |  |
| 4 | *K. pneumoniae* | 19 (5.5) | 25 (7.9) | 2.4% | 0.224 |  |
| 5 | *Acinetobacter* spp.^b^ | 9 (2.6) | 11 (3.5) | 0.9% | 0.522 |  |
| 6 | *S. pneumoniae* | 14 (4.1) | 6 (1.9) | -2.2% | 0.102 |  |
| 7 | *Enterobacter* spp. | 12 (3.5) | 6 (1.9) | -1.6% | 0.208 |  |
| 8 | Group B *Streptococcus* | 8 (2.3) | 6 (1.9) | -0.4% | 0.699 |  |
| 9 | *P. aeruginosa* | 6 (1.7) | 6 (1.9) | 0.1% | 0.887 |  |
| 10 | *E. faecalis* | 7 (2) | 3 (0.9) | -1.1% | 0.252 |  |
|  | Others | 162 (47.0) | 112 (35.2) |  |  |  |
| Adult | | |  |  |  |  |
| 1 | *E. coli* | 664 (29.6) | 1072 (32.5) | 2.9% | 0.022 | Increase |
| 2 | *K. pneumoniae* | 344 (15.3) | 493 (14.9) | -0.4% | 0.695 |  |
| 3 | *S. aureus* | 327 (14.6) | 448 (13.6) | -1.0% | 0.297 |  |
| 4 | *P. aeruginosa* | 92 (4.1) | 169 (5.1) | 1.0% | 0.077 |  |
| 5 | *Enterobacter* spp. | 90 (4) | 114 (3.5) | -0.6% | 0.282 |  |
| 6 | *Acinetobacter* spp.^b^ | 90 (4) | 110 (3.3) | -0.7% | 0.185 |  |
| 7 | *E. faecalis* | 49 (2.2) | 53 (1.6) | -0.6% | 0.117 |  |
| 8 | *Salmonella* spp. | 42 (1.9) | 53 (1.6) | -0.3% | 0.456 |  |
| 9 | *E. faecium* | 9 (0.4) | 52 (1.6) | 1.2% | <0.001 | Increase |
| 10 | Group B *Streptococcus* | 38 (1.7) | 48 (1.5) | -0.2% | 0.481 |  |
|  | Others | 500 (22.3) | 688 (20.8) |  |  |  |
| Elderly |  |  |  |  |  |  |
| 1 | *E. coli* | 946 (30.3) | 1652 (33.9) | 3.6% | <0.001 | Increase |
| 2 | *S. aureus* | 445 (14.3) | 660 (13.5) | -0.7% | 0.356 |  |
| 3 | *K. pneumoniae* | 363 (11.6) | 556 (11.4) | -0.2% | 0.749 |  |
| 4 | *P. aeruginosa* | 139 (4.5) | 238 (4.9) | 0.4% | 0.382 |  |
| 5 | *Acinetobacter* spp.^b^ | 135 (4.3) | 153 (3.1) | -1.2% | 0.005 | Decrease |
| 6 | *Enterobacter* spp. | 82 (2.6) | 131 (2.7) | 0.1% | 0.875 |  |
| 7 | *E. faecalis* | 76 (2.4) | 123 (2.5) | 0.1% | 0.809 |  |
| 8 | *P. mirabilis* | 79 (2.5) | 109 (2.2) | -0.3% | 0.392 |  |
| 9 | Group B *Streptococcus* | 50 (1.6) | 94 (1.9) | 0.3% | 0.287 |  |
| 10 | *Salmonella* spp. | 52 (1.7) | 91 (1.9) | 0.2% | 0.512 |  |
|  | Other | 753 (24.1) | 1070 (21.9) |  |  |  |

^a^ The ranking order shown is based on the number of isolates in 2012-2020.

^b^ *Acinetobacter* spp., here refers to *A. baumannii,* *A. nosocomialis*, and *A. pittii*, 3 species of the previously named *A. calcoaceticus-A. baumannii* complex.

Supplementary Table S4. Antimicrobial non-susceptibility (%) among Gram-negative bacteria causing bacteremia in Taiwan, 2002-2020

| Pathogens | 2002 | 2004 | 2006 | 2008 | 2010 | 2012 | 2014 | 2016 | 2018 | 2020 | 2002-2020 | P value | Trend  Direction |
| --- | --- | --- | --- | --- | --- | --- | --- | --- | --- | --- | --- | --- | --- |
| *E. coli* (N=4431) | | | | | | | | | | | | | |
| Cefazolin | 45.6 | 59.5 | 50.2 | 53.9 | 60.9 | 69.6 | 60.0 | 67.7 | 80.0 | 68.6 | 63.6 | <0.001 | Increase |
| Cefuroxime | 14.0 | 21.8 | 13.3 | 23.3 | 22.9 | 32.6 | 29.0 | 35.1 | 32.4 | 38.2 | 28.3 | <0.001 | Increase |
| Cefotaxime | 12.0 | 17.7 | 10.8 | 19.5 | 19.2 | 28.2 | 26.2 | 30.9 | 30.3 | 34.1 | 24.9 | <0.001 | Increase |
| Cefepime | 3.6 | 3.6 | 2.5 | 4.6 | 7.2 | 12.6 | 11.3 | 17.5 | 16.4 | 19.7 | 11.4 | <0.001 | Increase |
| Piperacillin-tazobactam | NT^a^ | NT | 3.3 | 5.5 | 4.1 | 4.7 | 4.6 | 6.3 | 4.5 | 5.9 | 5.0 | 0.451 |  |
| Imipenem | 0 | 0.5 | 0 | 0.8 | 0 | 0 | 0 | 0.3 | 0 | 1.8 | 0.4 | 0.01 | Increase |
| Trimethoprim/sulfamethoxazole | 59.6 | 56.8 | 51.6 | 51.2 | 46.4 | 55.2 | 47.7 | 46.2 | 47.5 | 40.8 | 49.2 | <0.001 | Decrease |
| Amikacin | 9.2 | 13.2 | 11.8 | 8.8 | 8.4 | 8.4 | 8.5 | 13.8 | 6.2 | 7.9 | 9.3 | 0.127 |  |
| Ciprofloxacin | 20.0 | 27.3 | 24.0 | 29.4 | 27.4 | 37.5 | 40.0 | 40.1 | 39.0 | 43.4 | 34.8 | <0.001 | Increase |
| *K. pneumoniae* (N=1813) | | | | | | | | | | | | | |
| Cefazolin | 15.6 | 29.5 | 23.3 | 28.6 | 29.9 | 27.1 | 29.4 | 35 | 44.4 | 35.2 | 30.8 | <0.001 | \| Increase \| \| --- \| |
| Cefuroxime | 5.5 | 16.4 | 15.5 | 19.6 | 20.6 | 20.6 | 23.8 | 23.5 | 26.3 | 72.8 | 26.4 | <0.001 | Increase |
| Cefotaxime | 3.1 | 14.8 | 12.6 | 16.6 | 19.1 | 20.2 | 22.4 | 21.2 | 22.4 | 25.8 | 19.0 | <0.001 | Increase |
| Cefepime | 0 | 0 | 0 | 0 | 2.6 | 8.3 | 11.7 | 10.1 | 7.8 | 15.0 | 6.6 | <0.001 | Increase |
| Piperacillin-tazobactam | NT | NT | 9.7 | 14.1 | 11.9 | 10.6 | 15.0 | 14.7 | 12.2 | 15.0 | 13.1 | 0.286 |  |
| Imipenem | 0 | 1.6 | 1.9 | 2.5 | 8.2 | 0.5 | 4.2 | 7.4 | 1.0 | 4.7 | 3.5 | 0.038 | Increase |
| Trimethoprim/sulfamethoxazole | 14.8 | 26.2 | 20.4 | 23.6 | 22.2 | 25.2 | 27.6 | 27.6 | 27.8 | 28.2 | 25 | 0.004 | Increase |
| Amikacin | 3.1 | 9.0 | 8.7 | 12.6 | 11.9 | 5.5 | 6.5 | 3.7 | 1.0 | 2.8 | 6.3 | 0.001 | Decrease |
| Ciprofloxacin | 8.6 | 17.2 | 15.5 | 21.1 | 27.3 | 24.8 | 30.8 | 28.6 | 29.8 | 32.4 | 25.1 | <0.001 | Increase |
| *Enterobacter* spp. (N=433) | | | | | | | | | | | | | |
| Cefotaxime | 34.4 | 38.5 | 17.6 | NT | 27.5 | 31.1 | 25.5 | 34.2 | 40.0 | 32.1 | 31.1 | 0.513 |  |
| Cefepime | 9.4 | 3.8 | 2.9 | 0 | 5.0 | 1.6 | 4.3 | 5.3 | 11.1 | 7.1 | 4.8 | 0.237 |  |
| Piperacillin-tazobactam | NT | NT | 8.8 | 5.6 | 15.0 | 19.7 | 8.5 | 21.1 | 26.7 | 21.4 | 16.0 | <0.001 | Increase |
| Imipenem | 12.5 | 15.4 | 14.7 | NT | 2.5 | 3.3 | 0 | 10.5 | 6.7 | 14.3 | 8.2 | 0.778 |  |
| Trimethoprim/sulfamethoxazole | 28.1 | 26.9 | 8.8 | 16.7 | 25.0 | 11.5 | 12.8 | 18.4 | 20.0 | 12.5 | 17.1 | 0.171 |  |
| Amikacin | 28.1 | 19.2 | 14.7 | NT | 7.5 | 3.3 | 0 | 2.6 | 4.4 | 3.6 | 7.9 | <0.001 | Decrease |
| Ciprofloxacin | 21.9 | 15.4 | 11.8 | NT | 17.5 | 21.3 | 14.9 | 13.2 | 26.7 | 21.4 | 19.2 | 0.442 |  |
| Non typhoidal *Salmonella*, NTS (N=372)^b^ | | | | | | | | | | | | | |
| 3GC^c^ | 0 | NT | 0 | 0 | 2.7 | 1.9 | 3.6 | 0 | 11.9 | 9.8 | 3.5 | 0.001 | Increase |
| Ciprofloxacin | 36.2 | NT | 28.6 | 14.7 | 13.5 | 11.3 | 14.5 | 14.3 | 16.7 | 12.2 | 17.7 | 0.004 | Decrease |
| *P. aeruginosa* (N=659) | | | | | | | | | | | | | |
| Ceftazidime | 6.0 | 7.9 | 14.8 | 9.3 | 14.8 | 8.8 | 15.2 | 10.3 | 13.4 | 10.0 | 11.1 | 0.387 |  |
| Cefepime | 8.0 | 7.9 | 7.4 | 6.7 | 14.8 | 7.7 | 15.2 | 7.7 | 9 | 6.3 | 9.3 | 0.998 |  |
| Piperacillin-tazobactam | 8.0 | 7.9 | 22.2 | 14.7 | 19.7 | 11.0 | 16.3 | 17.9 | 11.9 | 10.0 | 13.8 | 0.845 |  |
| Imipenem | 10.0 | 21.1 | 11.1 | NT | 16.4 | 7.7 | 22.8 | 6.4 | 19.4 | 17.5 | 15.3 | 0.441 |  |
| Amikacin | 4.0 | 2.6 | 3.7 | 1.3 | 6.6 | 1.1 | 2.2 | 0 | 3.0 | 3.8 | 2.6 | 0.651 |  |
| Ciprofloxacin | 18.0 | 21.1 | 29.6 | 17.3 | 19.7 | 15.4 | 17.4 | 19.2 | 17.9 | 10.0 | 17.5 | 0.145 |  |
| *Acinetobacter* spp. (N=514)^d^ | | | | | | | | | | | | | |
| Ceftazidime | 45.5 | 60.7 | 44.1 | 48.2 | 45.0 | 44.7 | 29.5 | 22.4 | 17.9 | 47.1 | 39.5 | <0.001 | Decrease |
| Cefepime | 42.4 | 50.0 | 41.2 | 45.8 | 41.7 | 44.7 | 24.6 | 34.7 | 21.4 | 44.1 | 38.5 | 0.012 | Decrease |
| Piperacillin-tazobactam | 48.5 | 71.4 | 41.2 | 48.2 | 45.0 | 47.4 | 37.7 | 32.7 | 30.4 | 50.0 | 44.0 | 0.010 | Decrease |
| Imipenem | 3.0 | 7.1 | 23.5 | 30.1 | 36.7 | 46.1 | 34.4 | 28.6 | 30.4 | 50.0 | 31.5 | <0.001 | Increase |
| Amikacin | 36.4 | 53.6 | 35.3 | 39.8 | 35.0 | 42.1 | 31.1 | 30.6 | 14.3 | 47.1 | 35.6 | 0.042 | Decrease |
| Ciprofloxacin | 45.5 | 64.3 | 50.0 | 48.2 | 45.0 | 48.7 | 37.7 | 36.7 | 21.4 | 50.0 | 43.6 | 0.005 | Decrease |

^a^ NT, not tested.

^b^ Antimicrobial susceptibility testing was not done on isolates from 2004. A total of 332 NTS isolates were analyzed here.

^c^ 3GC analysis for *Salmonella* spp., was based on cefotaxime in 2002, 2010, 2018, in 2020, on ceftazidime in 2006, 2008, 2016, and on ceftriaxone in 2012 and 2014.

^d^ *Acinetobacter* spp., here refers to *A. baumannii,* *A. nosocomialis*, and *A. pittii*, 3 species of the previously named *A. calcoaceticus-A. baumannii* complex.

Supplementary Table S5. Antimicrobial non-susceptibility (%) among Gram-positive bacteria causing bacteremia in Taiwan, 2002-2020

| Pathogens | 2002 | 2004 | 2006 | 2008 | 2010 | 2012 | 2014 | 2016 | 2018 | 2020 | 2002-2020 | P value | Trend  Direction |
| --- | --- | --- | --- | --- | --- | --- | --- | --- | --- | --- | --- | --- | --- |
| *S. aureus* (N=1932) | | | | | | | | | | | | | |
| Oxacillin | 41.6 | 51.9 | 52.0 | 56.1 | 55.8 | 51.3 | 55.6 | 48.1 | 52.1 | 44.6 | 51.2 | 0.902 |  |
| Clindamycin | 41.6 | 46.5 | 47.2 | 48.8 | 38.5 | 38.3 | 36.4 | 23.3 | 22.3 | 18.7 | 35.5 | <0.001 | Decrease |
| Erythromycin | 50.9 | 58.9 | 53.7 | 57.6 | 51.4 | 49.4 | 52.4 | 49.5 | 54.3 | 54.4 | 52.9 | 0.612 |  |
| Rifampin | 8.1 | 14.7 | 7.3 | 14.1 | 15.4 | 12.6 | 7.6 | 3.9 | 3.2 | 4.7 | 9.2 | <0.001 | Decrease |
| Trimethoprim/sulfamethoxazole | 29.2 | 30.2 | 27.6 | 28.3 | 25.5 | 23.4 | 16.4 | 12.6 | 10.6 | 8.8 | 20.6 | <0.001 | Decrease |
| Tetracycline | 55.3 | 58.1 | 51.2 | 55.1 | 61.1 | 50.6 | 38 | 42.2 | 35.6 | 31.6 | 47.3 | <0.001 | Decrease |
| Moxifloxacin | NT^a^ | NT | NT | 38.5 | 36.5 | 35.7 | 15.6 | NT | 37.2 | 34.2 | 32.6 | 0.087 |  |
| Quinupristin/dalfopristin | 0 | 0 | 0.8 | 0 | 0 | 0.4 | 1.6 | 0.5 | 0.5 | NT | 0.5 | 0.11 |  |
| Daptomycin | NT | NT | 0.8 | 0 | 1.9 | 0 | 0 | 1.5 | 0 | 0 | 0.5 | 0.321 |  |
| Vancomycin | 0 | 0 | 0 | 0 | 0.5 | 0 | 0 | 0 | 0 | 0 | 0.1 | 0.755 |  |
| *E. faecalis* (N=485) | | | | | | | | | | | | | |
| Ampicillin | 0 | 0 | 0 | 0 | 0 | 0 | 0 | 2.2 | 2.0 | 0 | 0.4 | 0.134 |  |
| Vancomycin | 0 | 0 | 3.2 | 1.6 | 0 | 0 | 0 | 0 | 0 | 0 | 0.4 | 0.236 |  |
| Linezolid | 0 | 0 | 0 | 0 | 0 | 2.7 | 0 | 11.1 | 12.0 | 11.9 | 3.7 | <0.001 | Increase |
| Daptomycin | NT | NT | 3.2 | 0 | 3.3 | 0 | 0 | 8.9 | 12.0 | 0 | 3.1 | 0.041 | Increase |
| *E. faecium* (N=278) | | | | | | | | | | | | | |
| Vancomycin | 0 | 10.0 | 0 | 14.3 | 15.4 | 36.4 | 38.2 | 40.5 | 66.7 | 47.4 | 35.6 | <0.001 | Increase |
| Linezolid | 0 | 0 | 0 | 0 | 0 | 0 | 0 | 0 | 4.8 | 2.6 | 1.1 | 0.06 |  |
| Daptomycin | NT | NT | 0 | 0 | 0 | 2.3 | 2.9 | 5.4 | 2.4 | 2.6 | 2.3 | 0.269 |  |
| Group B *Streptococcus* (N=570) | | | | | | | | | | | | | |
| Clindamycin | NT | NT | NT | NT | 41.7 | 41.7 | 44.4 | 23.8 | 30.6 | 34.9 | 36.8 | 0.047 | Decrease |
| Erythromycin | 28.6 | 40.9 | 77.8 | 45.0 | 47.6 | 45.8 | 44.4 | 31.3 | 34.7 | 39.8 | 41.4 | 0.128 |  |
| Levofloxacin | 0 | 0 | 11.1 | 5.0 | 6.0 | 1.0 | 3.3 | 5.0 | 5.6 | 4.8 | 4.0 | 0.383 |  |
| Tetracycline | 92.9 | 100 | 88.9 | 85 | 78.6 | 70.8 | 71.1 | 82.5 | 73.6 | 78.3 | 77.5 | 0.067 |  |
| Penicillin | 0 | 0 | 0 | 0 | 0 | 0 | 0 | 0 | 0 | 0 | 0 | - |  |

^a^NT, not tested.
